# A New Horizon of Liquid Biopsy in Thymic Epithelial Tumors: The Potential Utility of Circulating Cell-Free DNA

**DOI:** 10.3389/fonc.2020.602153

**Published:** 2021-02-04

**Authors:** Margaret Ottaviano, Mario Giuliano, Marianna Tortora, Evelina La Civita, Antonietta Liotti, Michele Longo, Dario Bruzzese, Michele Cennamo, Vittorio Riccio, Pietro De Placido, Fernanda Picozzi, Sara Parola, Bruno Daniele, Gerardo Botti, Pietro Formisano, Francesco Beguinot, Sabino De Placido, Daniela Terracciano, Giovannella Palmieri

**Affiliations:** ^1^ Department of Clinical Medicine and Surgery, Università degli Studi di Napoli “Federico II”, Naples, Italy; ^2^ CRCTR Rare Tumors Coordinating Center of Campania Region, Naples, Italy; ^3^ Oncology Unit, Ospedale del Mare, Naples, Italy; ^4^ Department of Translational Medical Sciences, Università degli Studi di Napoli “Federico II”, Naples, Italy; ^5^ Department of Public Health, Università degli Studi di Napoli “Federico II”, Naples, Italy; ^6^ Pathology Unit, Istituto Nazionale Tumori-IRCCS-Fondazione G. Pascale, Naples, Italy

**Keywords:** thymic epithelial tumors, circulating cell-free DNA, biomarkers, stage system, circulating tumor DNA, thymoma, thymic carcinoma

## Abstract

**Background:**

Thymic epithelial tumors (TETs) are rare thoracic malignancies, commonly divided into two different histopathological entities, thymoma (T) and thymic carcinoma (TC). To date, there are no specific biomarkers for monitoring the biological course of these rare tumors. We carried out a single center study aiming at the detection of circulating cell-free DNA (ccfDNA) and the correlation of its levels with metastatic dissemination and histological subtype in patients with TETs.

**Methods:**

From July 2018 to January 2020, 5-ml blood samples from 26 patients with advanced TET (aTET) (11 patients with TC and 15 patients with T) and from six patients with completely resected TET (cr-TET), were prospectively obtained before the initiation of systemic therapy. Blood samples from 10 healthy donors were used as control. The QIAamp MinElute ccfDNA Kits was used for ccfDNA isolation from plasma; real-time PCR was used for cfDNA quantification.

**Results:**

We found significantly higher ccfDNA amount in patients with T and TC compared to controls, with median ccfDNA level of 3.3 ng/µl, 11.4 ng/µl and 25.6 ng/µl, for healthy donors, T and TC patients, respectively (p<0.001). No significant difference was found between cr-TET and controls (p = 0.175). ccfDNA concentrations were higher in metastatic (M1a and M1b) compared to non-metastatic (M0) TETs (25.6 ng/µl versus 7.2 ng/µl; p= 0.037). No significant correlation was found either between ccfDNA and disease stage, according to both the Masaoka-Koga (p= 0.854) and the TNM 8th edition staging systems (p = 0.66), or between ccfDNA levels and overall tumor burden, estimated according RECIST 1.1 criteria (r = 0.07, p = 0.725).

**Conclusions:**

To the best of our knowledge, this is the first study that prospectively explores detection and quantification of ccfDNA in TETs. Higher baseline cfDNA levels have been observed in both advanced T and TC comparing to the control group.

## Introduction

Thymic epithelial tumors (TETs) are rare thoracic malignancies. Widely recognized as morphologically different, thymoma (T) and thymic carcinoma (TC) show also a different biological behavior with higher tendency to hematogenous dissemination and aggressive biological course for TC, and thoracic recurrence with uncertain or definitely malignant biological behavior for T (1–3). These complex histopathological entities share a poor prognosis when characterized by high tumor burden and distant metastases (4). As matter of fact, staging at diagnosis is widely recognized as the major prognostic factor (5, 6), whereas histotype classification has still a debated prognostic relevance. Moreover, the unpredictable oncological outcomes of TETs, the frequent association with immunological dysregulations and the preservation of variably thymopoietic activity, make these rare malignancies really challenging to manage (7, 8). Despite the lack of large phase II and III clinical trial investigations, and the slow progresses of new drug development, the treatment strategies and the molecular profile knowledge of newly diagnosed and unresectable/metastatic/recurrent TETs have evolved over time (9). Indeed, recurrent gene mutations for both T and TC have been identified (10), and pathogenetic mechanisms underlying the association between TETs and autoimmune diseases have been deeply investigated (11–13). In addition, promising data about the activity of targeted therapies and immune checkpoints inhibitors (ICIs) have recently emerged (14–17). However, there is still major uncertainty regarding the oncogenic potential of these rare neoplasms and it is unclear whether a deeper molecular characterization, with the identification of novel biomarkers, could bring to relevant progresses in clinical management of TETs, significantly improving their diagnosis, treatment, and follow up strategies, and ultimately their prognosis.

Liquid biopsy approaches, especially those involving the isolation of cell-free DNA (cfDNA) from plasma, have recently emerged as a useful and minimally invasive tool for molecular analysis, for exploring tumor heterogeneity, and for detecting and monitoring cancer through its biological course (18). The term cell-free DNA was first reported in 1948 by Mandel and Metais, referring to fragmented DNA detected in the non-cellular component of the blood. In healthy persons normal cells release in plasma low levels of cfDNA (approximately 10 to 15 ng/ml), that can raise in particular circumstances of tissue stress, such as exercise, inflammation, surgery, or tissue injury (19). Patients with cancer are likely to have higher overall levels of cfDNA than healthy persons, as already observed more than 40 years ago. The term circulating tumor DNA (ctDNA) refers to cfDNA, released into plasma from tumor cells in a state of apoptosis or necrosis and represents only a part of the overall cfDNA. The term ctDNA is usually used to define short DNA fragments (<166 pb) and the fraction of ctDNA in patients with tumor can differ greatly, from less than 0.1% to more than 90%. Although the fraction of ctDNA seems to be related to tumor burden within an individual patient, heterogeneity has been discovered among patients with the same tumor type, conceivably reflecting biologic differences or differences in rates of cell death in those specific tumors (20–22).

Several studies showed that ctDNA is a promising biomarker potentially useful for early diagnosis and detection of relapse, for prognostic assessment, as well as for evaluation of treatment response in a variety of malignancies, such as breast, colon and lung cancer. Importantly, due to its short half-life (about 2 h), it is recognized that ctDNA analysis may be representative of the real-time molecular changes in the tumor (23–35). However, there is still lack of evidence regarding the role of cfDNA or ctDNA analysis in rare tumors.

For the first time, we explored the detection and the correlation of ccfDNA levels with histological and staging features of TETs, in a single center study, in order to identify a novel, reproducible and non-invasive biomarker in these rare malignancies.

## Materials and Methods

### Study Design and Participants

This was a prospective study evaluating the detection and the clinical utility of cfDNA in a series of patients with TETs, conducted at a single academic center, the Rare Tumors Coordinating Center of Campania Region (CRCTR) at University of Naples Federico II. Starting from July 2018 to January 2020, plasma samples were prospectively obtained after at least 3 months from surgery from 6 patients with completely resected TETs (crTETs), and before initiation of systemic therapy from 26 patients with advanced TETs (aTETs), comprising *de novo* metastatic/unresectable TETs and progressing/relapsing TETs. aTETs consisted of 15 patients with T and 11 with TC. Only patients able to safely stop immunosuppressive therapy were included in our study and underwent blood sample collection. Immunosuppressive treatments (including steroids) for autoimmune diseases control were stopped at least four weeks before peripheral blood sample collection. Plasma samples of 10 healthy donors were used as control. Histo-pathological features according to World Health Organization (WHO) Histological Classification of 2015 (1–3) and stage disease according to both the Masaoka-Koga and TNM8th edition staging systems (6, 36, 37) were assessed. The overall baseline tumor burden of aTETs patients was calculated according to RECIST (Response Evaluation Criteria In Solid Tumors) criteria version 1.1. All the patients underwent total body CT scan with iv contrast at baseline, before the blood collection. A sum of the diameters (longest for non-nodal lesions, short axis for nodal lesions) for all target lesions up to a maximum of five total lesions (and a maximum of two lesions per organ) representative of all involved organs, was calculated and reported as the baseline sum diameters. The study was approved by the Ethical Committee of University of Naples Federico II and all the enrolled patients provided informed consent.

### Circulating Cell-Free DNA Extraction

Blood was sampled in EDTA collection tubes and processed within an hour. The plasma was obtained through two centrifugations: 1600 rcf and 16,000 rcf, both at 4°C for 10 min. 4 ml aliquots of plasma were stored at −80°C. The isolation of cfDNA from human plasma (2 ml) was performed with QIAamp MinElute ccfDNA Kits (ref. 55204, Qiagen) according to the manufacturer’s protocol. Columns were eluted with 40 μl of free nuclease water. Real-time PCR quantitative assay was designed to quantify the amount of cfDNA in each sample against a standard curve. Two different primer sets were designed using the β-globin (BGLO) (GeneBank accession number: U01317) as the reference gene, BGLO40 for the 40 bp amplicon and BGLO300 for the 300 bp amplicon. The Ct value obtained in BGLO40 PCR reaction was used for the ctDNA absolute quantification, while BGLO300 was used to evaluate DNA integrity ratio as further described below.

The sequences of BGLO40 primers were: F-GCTCCACAGGGTGAGGTCTAA -, R-CAGGTACGGCTGTCATCACTT -; for the BGLO300 the sequences were: F-GCTCCACAGGGTGAGGTCTAA -, R-ACATATCCCAAAGCTGAATTATGGT-.

In detail, for each sample, reactions were performed in duplicates using iQ SYBR Green Supermix (ref. 1708886 Bio-rad laboratories) on a QuantStudio 7 Flex Real-time PCR detection system (ref. 4485701 ThermoFischer) by using the following cycling condition and reaction protocol. Cycling condition: 1 min at 95°C for 1 cycle, and 5 s at 95°C and 30 s at 60°C repeated for 40 cycles. Reaction protocol: cfDNA (2 µl of eluted sample), forward and reverse PCR primers (200 nM each), iQ SYBR Green Supermix 1× in a final volume of 10 μL (38).

The absolute concentration of the cfDNA was calculated using a standard curve with serial dilutions (15 ng – 0.01 pg) of genomic DNA obtained from peripheral blood leukocytes as external standard.

Each point of the standard curve was quantified in triplicate and were included in each run (only assays with R2 values above 0.99 for the standard curve have been accepted). A negative control was performed in each plate. Repeatability and reproducibility were determined by repeated measurement of the same sample. Repeatability was evaluated by the correlation between quantification values obtained from independent tests. The real-time PCR quantitative inter-assay repeatability was evaluated on the same ccfDNA samples by the same observer under identical conditions, in a short period of time. The real-time PCR quantitative assay reproducibility was evaluated by running the test on the same ccfDNA samples in 2 different laboratories.

### DNA Integrity Ratio

We used real-time PCR to quantify the amount of cfDNA against standard curves, using two amplicons of different lengths in the same β-globin gene: 40 bp, and 300 bp (forward primer is in common in both reactions). DNA integrity ratio was calculated as the ratio of BGLO300-qPCR to BGLO40-qPCR and it is used as a quality control test. The evaluation of integrity ratio allowed to detect human genomic DNA contamination in cfDNA samples. In accordance with Nikolaev S et al., ctDNA has an average length of about 170 bp, and amplification would perform poorly for the 300 bp amplicon (39). Conversely, in case of contamination by genomic DNA of leukocytic origin, amplification of larger amplicons ought to be proficient and DNA integrity ratio is ∼0.85 (39). Our cfDNA samples showed a median of DNA integrity ratio of 0,088 (IQR: 0.001–0.204), suggesting that contamination by genomic DNA can be considered irrelevant and most probably we detected ctDNA.

### Statistical Analysis

Quantitative variables were reported as mean ± standard deviation (SD), and variables with skewed distributions presented as median and range (min to max). Categorical variables were summarized and reported as frequencies and percentages. Accordingly, between groups comparison were based on the student T test, the Mann Whitney U test and the Fisher exact test. In case of three groups, Kruskal-Wallis nonparametric ANOVA was used as omnibus test followed by Mann Whitney test for pairwise comparison and p-values were adjusted using Holm procedure. Correlation among numerical variables was assessed using Spearman correlation coefficient. Statistical significance was set at two sided p value<0.05. All analyses were performed using the statistical platform R (vers. 3.5.2).

## Results

### Study Population

The characteristics of enrolled patients with aTETs and crTETs are summarized in **Tables 1** and **2**, respectively. The Characteristics of healthy controls are reported in the **Table S1** in the appendix section.

**Table 1:** Characteristics of 26 patients with aTETs (15 T and 11 TC).

Characteristics	Values (%)
Age, mean (median)± SD	59 (59.5) ± 10.33
Range	37–76 years
Sex	
Male	14 (53.8%)
Female	12 (46.2%)
Histologic Type	
Thymoma	15 (57.7%)
A	2 (13.3%)
AB	1 (6.6%)
B1	1 (6.6%)
B2	3 (20.0%)
B1-B2	1 (6.6%)
B2-B3	3 (20.0%)
B3	3 (20.0%)
NOS	1 (6.6%)
Thymic carcinoma	11 (42.3%)
SCC	9 (81.8%)
LLCC	1 (9.1%)
NOS	1 (9.1%)
Radiological Stage of Disease according to TNM	
III	4 (15.4%)
IVA	10 (38.6%)
IVB	12 (46%)
Radiological Stage of Disease according to Masaoka-Koga	
III	2 (7.7%)
IVA	14 (53.8%)
IVB	10 (38.5%)
Autoimmunity	
Yes	12 (46.2%)
No	14 (53.8%)
Autoimmune Disease	
Myasthenia Gravis	3 (11.5%)
Isaacs Syndrome	1 (3.8%)
Hashimoto Thyroiditis	4 (15.4%)
Basedow-Graves’ Disease	2 (16.6%)
Crohn’s Disease	2 (16.6%)

NOS, not otherwise specified; SCC, squamous cell carcinoma; LLCC, lymphoepithelioma like cell carcinoma.

**Table 2:** Characteristics of 6 patients with crTETs.

Characteristics	Values (%)
Age, mean (median)± SD	45.6 (45.6) ± 9.33
Range	36–57 years
Sex	
Male	0
Female	6 (100%)
Thymoma	5 (83.3%)
Thymic Carcinoma (SCC)	1 (14.3%)
Radiological Stage of Disease according to TNM	
I	3 (50.0%)
II	2 (38.6%)
IIIb	1 (16.7%)
Radiological Stage of Disease according to Masaoka-Koga	
I	3 (50.0%)
IIa	2 (33.3%)
III	1 (38.5%)
Pathological Stage of Disease according TNM	
Stage I	3 (50.0%)
Stage II	2 (33.3%)
Stage IIIa	1 (16.7%)
Pathological Stage of Disease according to Masaoka-Koga	
Stage I	3 (50.0%)
Stage IIa	2 (33.3%)
Stage III	1 (16.7%)
Autoimmunity	
Yes	3 (50.0%)
No	3 (50.0%)
Autoimmune Disease	
Myasthenia Gravis	2 (33.3%)
Hashimoto Thyroiditis	1 (16.64%)

SCC, squamous cell carcinoma.

Twenty-six patients (11 Thymic Carcinoma and 15 Tymomas) with aTET, 6 patients with crTET, and 10 controls were enrolled. Healthy controls were slightly younger than TET patients (51.2 ± 8.7 vs. 59.6 ± 10.3) with no difference in the male/female ratio (14/12 vs 5/5; p=1). For thymoma histology all the known subtypes were included in the series (A, AB, B1, B2, B1-B2, B2-B3, B3). Regarding TC, all the cases included in the series were squamous cell carcinoma (SCC) subtypes, with the exception of one case of lymphoepithelioma like cell carcinoma (LLCC) and one case of not otherwise specified carcinoma (NOS). The majority of patients had metastatic disease with prevalent stages IVA and IVB according to both stage systems. Of note, two patients with TNM stage IIIB disease were classified as stage IVA according to the Masaoka-Koga staging system. Autoimmune diseases were present in 12 patients (46% of aTET patients), of whom 4 suffered from neurological immune dysregulation (all the autoimmune diseases are listed in **Tables 1** and **2**). The median age for crTETs was 44 years (range: 3–57), one patient had thymic carcinoma, whereas the remaining 5 patients had thymoma. Autoimmune diseases were detected in 50% of crTETs patients, of whom 2 had myasthenia gravis.

### ccfDNA Detection in aTETs, crTETs, and Controls

**Figure 1:**
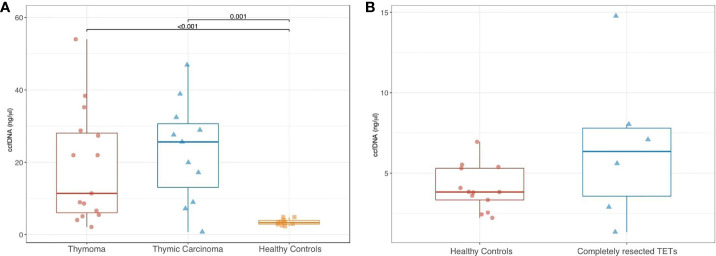
Comparison of ccfDNA levels in patients with thymoma vs thymic carcinoma vs healthy controls **(A)**, and in patients with completely resected TETs vs healthy controls **(B)**.

Among patients with aTETs the median quantity of ccfDNA measured prior to starting systemic treatment was respectively 11.4 ng/µl (range, 2.1–54) and 25.6 ng/uL (range 0.8- 46.9), for T and TC. A median ccfDNA value of 3.3 (range, 2.3–4.9) was detected in healthy controls **(** **Tables S2**, **S3** in the Appendix section). Analysis of ccfDNA concentrations in aTET patients compared to healthy subjects revealed statistically significant differences (11.4 and 25.6 for T and TC patients, respectively *versus* 3.3 ng/µl for healthy donors; p=0.001 and p<0.001). No statistically significant difference was found comparing ccfDNA median values in T versus those observed in TC patients (p= 0.384) **(** **Figure 1A** **)**. Median ccfDNA measured in crTET patients was 6.3 ng/uL (range 1.3–14.8) and no statistically difference was detected in comparison to healthy controls (p= 0.175) **(** **Figure 1B** **) (** **Table S4** in the Appendix Section).

### Correlation of ccfDNA Amount With Disease Stage in aTET Patients

**Figure 2:**
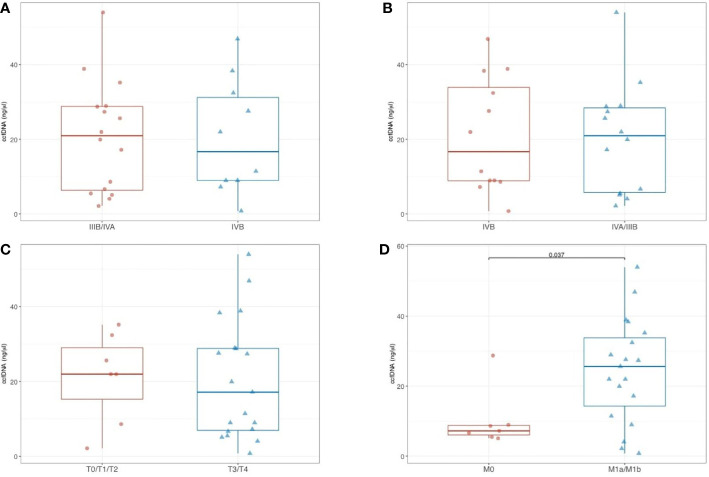
Comparison of ccfDNA levels in aTET patients according to clinical stage Masaoka-Koga **(A)**, and according to TNM 8^th^ edition **(B)**; comparison of ccfDNA levels according with single components of TNM staging system (T0/T1/T2 vs T3/T4) **(C)**; comparison of ccfDNA levels in M1a/M1b aTETs vs M0 aTETs **(D)**.

No significant difference in ccfDNA concentrations was found among aTET patients according to clinical stage Masaoka-Koga (III+IVA versus IVB; p= 0.854) (**Figure 2A** **)**. The same was observed according to TNM 8^th^ edition staging system (IIIB+IVA versus IVB; p= 0.662) **(** **Figure 2B** **).** When correlated with single components of TNM staging system, no significant difference (p = 0.862) was shown between T0/T1/T2 tumors (median ccfDNA = 22 ng/µl; range, 2.1–35.2) and T3/T4 tumors (median ccfDNA = 17.2; range: 0.8–54) **(** **Figure 2C** **)**. In contrast, ccfDNA concentrations were higher in M1a/M1b aTETs compared to M0 aTETs (25.6 ng/µl versus 7.2 ng/µl, respectively; p= 0.037) **(** **Figure 2D** **).**

### Correlation of ccfDNA Amount With Tumor Burden According RECIST Criteria

Median overall tumor burden estimated with RECIST criteria v 1.1 was 104 mm for aTETs (90 mm and 107 mm for TC and T, respectively) **(** **Table S2** **)**. No significant correlation was found between ccfDNA quantification and overall tumor burden (r= 0.07, p= 0.725).

## Discussion

Thymic epithelial malignancies are widely recognized as a rare but well-established group of organ-specific neoplasms with complex histopathological and staging features and variable malignant potential. We prospectively detected ccfDNA in peripheral blood samples from 26 patients with *de novo* or relapsing/progressing aTETs and from 6 patients with crTETs referred to an Italian Reference Center for thymic malignancies over an 18-months period.

Importantly, our samples showed a particularly low median value of DNA integrity ratio, indicating that we most probably identified ctDNA.

The amount of detected ccfDNA showed a significant correlation with metastatic dissemination, according to TNM staging systems (M1a and M1b stages: separate pleural or pericardial nodule(s) and pulmonary intraparenchymal nodule or distant organ metastasis, respectively) (40). In contrast, no significant correlation was found with the degree of local invasion of mediastinal structures (from T1 to T4, according to TNM staging system) (41). Notably, no correlation was found between ccfDNA levels and overall stage, for either the Masaoka-Koga and TNM, which currently are the most used and widely recognized staging systems for TETs. Unexpectedly, no significant differences in median ccfDNA values were observed between T and TC, despite these two group of malignancies show different biological features, as widely recognized. This may suggest that ctDNA peripheral release is associated with high disease burden and advanced stage, rather than with biological disease aggressiveness, as shown in our series. Larger studies are needed in order to achieve a definitive conclusion. Moreover, we found no correlation of ccfDNA amount with overall tumor burden according to RECIST 1.1 criteria. This finding possibly support the existing scepticism about clinical utility of RECIST 1.1 criteria for TETs radiological assessment (42). Finally, patients with crTETs had no statistical difference in detectable ccfDNA compared to healthy controls, suggesting the potential value of using ccfDNA as an easy and reproducible biomarker to better define the absence of residual disease in patients with TETs who undergo surgery with radical intent. We warrant future larger prospective studies evaluating ccfDNA analysis as a tool for identification of minimal residual disease and early diagnosis of relapse in TET patients.

Our findings could have potential implications also for improved prognosis definition in TETs, which, to date, is still undefined and complex, being influenced by multiple tumor-related, patient-related, treatment-related, and environment-related factors (6). While ctDNA analysis has already been proven potentially useful for patients with high incidence and prevalence neoplasms (23–35), there have been relatively few studies evaluating the utility of ccfDNA or ctDNA as a prognostic biomarker in patients with rare tumors. Worthy of consideration are several recently published studies, that evaluated detection of ctDNA and mutational analysis in patients with soft tissue sarcoma (STS). Identification of cancer associated *TP53*/*PIK3CA* mutations in two patients’ plasma matching with primary tumor tissue was carried out using Ion AmpliSeq™ panel in a series of 11 patients with STS (43). In addition, in a series of liposarcomas, correlation of detectable ctDNA with the clinical evolution and tumor burden was observed in 4 patients with myxoid liposarcomas (44). Mutational analysis of detected ctDNA might impact especially in rare malignancies associated with recurrent driver genetic events, such as GIST, whose course of disease and relative management is strictly dependent on KIT or PDGFRA oncogenic activation. However, in a recent study evaluating ctDNA mutations in a subset of GIST patients with bulky disease, detected ctDNA levels appeared to be lower in GIST than in other neoplasms, maybe due to some intrinsic characteristics of GIST biology (45).

The main limitations of our study are the relative small sample size and the absence of mutational analysis of the identified ccfDNA. However, considering the rarity of thymic malignancies, we believe that our study represents a promising proof-of-principle report, demonstrating the feasibility of quantitative detection of ccfDNA and its correlation with metastatic dissemination, regardless the histological subtypes, in aTET patients. Furthermore, basing on our preliminary data, we foresee the launch of international partnerships [i.e. within the European Reference Network (ERN-EURACAN)] aiming at assessing the clinical utility of ccfDNA detection in larger cohort of TET patients and also evaluating the presence and clinical relevance of driver mutations, such as those recently discovered in TETs by Radovich et al. (10).

## Conclusion

To the best of our knowledge, this is the first study prospectively evaluating the detection and quantification of ccfDNA in TETs. Higher baseline levels than the control group were observed in both advanced T and TC patients. Highest levels of ccfDNA were associated with the presence of distant metastasis. We envision that further valuable information can be obtained by performing ctDNA mutational analysis, which could better clarify the role of ctDNA as novel and reproducible biomarker in thymic malignancies.

## Data Availability Statement

The raw data supporting the conclusions of this article will be made available by the authors, without undue reservation.

## Ethics Statement

The studies involving human participants were reviewed and approved by Ethical Committee of University Federico II of Naples, Naples, Italy. The patients/participants provided their written informed consent to participate in this study.

## Author Contributions

The following authors have made substantial contributions to the intellectual content of the paper in the various sections: Conception and design: MO, MG, DT, GP; acquisition of data: MO, MT, EC, AL, ML, MC, VR, PP, FP, SP, BD; analysis and interpretation of data: MO, MG, DT, GP; drafting of the manuscript: MO; critical revision of the manuscript for important intellectual content: MO, PF, FB, SP, MG, DT, GP, GB; statistical analysis: DB; administrative, technical, or material support: MO, MT, MG, DT, GP; supervision: DT, GP, MG. All authors contributed to the article and approved the submitted version.

## Conflict of Interest

The authors declare that the research was conducted in the absence of any commercial or financial relationships that could be construed as a potential conflict of interest.
