# Supplementary material for: A New Horizon of Liquid Biopsy in Thymic Epithelial Tumors: The Potential Utility of Circulating Cell-Free DNA
Source: Front Oncol. 2021 Feb 4;10:602153. doi: 10.3389/fonc.2020.602153 (PMC7902074; doi:10.3389/fonc.2020.602153)
Supplement: Supplementary file 1 [file DataSheet_1.docx]

# Supplementary Material

# Table S1: Characteristics of 10 healthy controls

| Characteristics | Values (%) |
| --- | --- |
| Age, mean (median)± SD  Range | 51.2 (51.5) ± 8.6  37-64 years |
| Sex  Male  Female | 5 (50%)  5 (50%) |

**Table S2: ccfDNA amount and Tumor burden evaluation for 26 patients with aTET**

| **ID Sample** | **Histo-pathology** | **Tumour assessment** | **ccfDNA (ng/**μ**l)** | **Tumor Burden (mm) RECIST 1.1** |
| --- | --- | --- | --- | --- |
| **2** | **Thymic Carcinoma** | **advanced** | 38,867 | 75 |
| **4** | **Thymic Carcinoma** | **advanced** | 27,582 | 70 |
| **15** | **Thymic Carcinoma** | **advanced** | 28,924 | 119 |
| **7** | **Thymic Carcinoma** | **advanced** | 32,416 | 63 |
| **28** | **Thymic Carcinoma** | **advanced** | 25,625 | 34 |
| **29** | **Thymic Carcinoma** | **advanced** | 0,764 | 182 |
| **20** | **Thymic Carcinoma** | **advanced** | 46,890 | 157 |
| **13** | **Thymoma B3/Thymic Carcinoma** | **advanced** | 19,930 | 77 |
| **31** | **Thymic Carcinoma** | **advanced** | 8,956 | 90 |
| **32** | **Thymic Carcinoma** | **advanced** | 7,213 | 249 |
| **36** | **Thymic Carcinoma** | **advanced** | 17,167 | 105 |
| **Median** |  |  | **25,6** | **90** |
| **1** | **Thymoma A** | **advanced** | 53,997 | 100 |
| **26** | **Thymoma B2** | **advanced** | 35,212 | 103 |
| **11** | **Thymoma B2-B3** | **advanced** | 38,352 | 391 |
| **9** | **Thymoma B1-B2** | **advanced** | 21,972 | 75 |
| **23** | **Thymoma B3** | **advanced** | 11,412 | 80 |
| **34** | **Thymoma B1** | **advanced** | 5,471 | 36 |
| **46** | **Thymoma B3** | **advanced** | 8,964 | 107 |
| **40** | **Thymoma B3** | **advanced** | 2,133 | 48 |
| **41** | **Thymoma A** | **advanced** | 21,960 | 101,5 |
| **42** | **Thymoma B2** | **advanced** | 4,041 | 158 |
| **47** | **Thymoma** | **advanced** | 28,755 | 131 |
| **51** | **Thymoma B2** | **advanced** | 6,638 | 122 |
| **53** | **Thymoma B2-B3** | **advanced** | 8,610 | 127 |
| **54** | **Thymoma B2-B3** | **advanced** | 27,363 | 117 |
| **55** | **Thymoma AB** | **advanced** | 5,087 | 150 |
| **Median** |  |  | **11,4** | **107** |

**Table S3: ccfDNA amount for 6 patients with crTET**

| **ID** | **Histo-pathology** | **Tumour assessment** | **ccfDNA (ng/**μ**l)** |
| --- | --- | --- | --- |
| **52** | **Thymoma** | **completely resected** | 8,040 |
| **17** | **Thymic Carcinoma** | **completely resected** | 1,346 |
| **27** | **Thymoma** | **completely resected** | 14,766 |
| **35** | **Thymoma** | **completely resected** | 7,090 |
| **43** | **Thymoma** | **completely resected** | 5,605 |
| **48** | **Thymoma** | **completely resected** | 2,900 |
| **Median** |  |  | **6,3** |

**Table S4: ccfDNA amount for 10 healthy controls**

| **ID Sample** | **Histo-pathology** | | **Tumour assessment** | | **ccfDNA (ng/**μ**l)** | |
| --- | --- | --- | --- | --- | --- | --- |
| **77** | **Healthy Control** | | **-** | | 2,877 | |
| **78** | **Healthy Control** | | **-** | | 3,351 | |
| **79** | **Healthy Control** | | **-** | | 2,993 | |
| **80** | **Healthy Control** | | **-** | | 2,539 | |
| **81** | **Healthy Control** | | **-** | | 4,845 | |
| **82** | **Healthy Control** | | **-** | | 2,327 | |
| **86** | **Healthy Control** | | **-** | | 4,854 | |
| **61** | **Healthy Control** | | **-** | | 4,080 | |
| **62** | **Healthy Control** | | **-** | | 3,340 | |
| **64** | **Healthy Control** | | **-** | | 3,6 | |
| **Median** | |  | |  | | **3,3** |
